# Abstract of the Proceedings of the Buffalo Medical Association

**Published:** 1871-10

**Authors:** W. C. Phelps

**Affiliations:** Secretary


ART. III.—*Abstract of the Proceedings of the Buffalo Medical Association.*

BUFFALO, October 3, 1871.

The President in the Chair.

DR. CRONYN reported a case of great interest to the profession —a gun-shot wound of the head, the ball being carried eight years in the brain. Eight years ago was called to attend a lad fourteen years of age, who had received a ball from a gun which entered at the internal angle of the left orbit, without injuring the eye, passed through the left orbital plate of the frontal bone and onwards into the brain. The ball could be followed with a probe directly backwards and felt where it had lodged, four and one-half inches from its point of entrance. The wound closed and the boy enjoyed very good health till six months ago, when, making an assault on a neighboring farmer, he was struck with a hoe on the head, which made a scalp wound from which there was considerable hemorrhage. The wound was properly dressed, but a short time after he was taken with convulsions, and died. A coroner's investigation was had and an autopsy made. The inner table of the skull was a little depressed and there was some effusion of serum and a little matter. The ball which he had received was found between the posterior lobes of the brain and the cerebellum at the tentorium cerebelli. There was no indication of the track through the brain and it was covered by a false membrane and suspended by a small pedicle. After receiving the gun-shot wound the patient was unconscious four days; then became conscious but could not speak; was also paralyzed for a time. After his recovery, so as to resume his studies at school, he was obliged to learn to read anew, though at the time of his injury he could read well, but his knowledge of figures was not the least impaired, being able to cypher as well as before or better. His disposition was changed, being very easily irritated and had little control over his passions.

Dr. ROCHESTER remarked that this was an interesting case, particularly at the present time, for a trial for murder is soon to occur here, that of Kelley for murdering Rosenfelt on Michigan street, in which the defense will claim that Kelley was not responsible for his actions. Some time ago it is said he received an injury of the head from a falling tree, and since that time when in liquor or excited he becomes wild and ungovernable. The point raised is an important one in its bearing in criminal cases and many members of this association will probably be called on to express their opinions on it.

Dr. LITTLE mentioned a case somewhat similar which occurred about two years ago. A passenger on one of the railway trains of this city suddenly became insane and stabbed two fellow passengers and then jumped into the canal. He made a post-mortem section of the head and found ulceration and suppuration of the brain.

Dr. MINER related the case of the young man Hawks, who accidently shot himself in the head about two years since. The ball is still in the head, and it appears from Dr. Cronyn's report may possibly be in the brain; though he had never believed it, but supposed it was turned from direct course and lodged in the bones of the face. Prof. Steele, of St. Louis, formerly Demonstrator of Anatomy here, has recently written a paper on diseases and injuries of the brain, in which he asserts that in all severe injuries of the brain the mind is more or less impaired. He mentions the well known case of the quarry-man through whose head the tamping iron was blown by an accidental discharge of blast, and states that contrary to all the reports which have been circulated, his mind was much impaired. He regarded Dr. Cronyn's case as having many points of great interest.

Dr. ROCHESTER reports the following case of placenta prævia. Six months ago I was engaged to attend a lady in her second confinement. Two years before, while in Brooklyn, she was delivered of her first child after a very severe labor, and some of her friends had doubts as to her being safely delivered a second time, though the patient herself shared none of these fears. Ten days before the labor came on I was called to see her on account of hemorrhage. Found the os slightly dilated, and though suspecting that I had a case of placenta prævia, did not deem it prudent to attempt to determine the point positively by examining the os internum. Owing to the thinness of the abdominal walls I was able to determine accurately the position of the child, but could not hear the placental souffle. This tended to confirm the opinion I had formed of the case. There was no more hemorrhage till labor occurred. On the night of the labor, I was called by her husband at 8 o'clock to attend her. Found the os a little dilated and soft. I at once applied the tampon very firmly and also a bandage over the fundus of the uterus; by these means having perfect control of hemorrhage. Sent for Dr. White as counsel. Gave her half grain of morphine. She was pale and very anxious owing to alarm, some one having told her that she had placenta prævia and would not recover. The pains continued rather strong and regular, though she fainted twice notwithstanding stimulants and nourishment were freely given. I could not understand why she should be so weak and faint and knowing that she would not live long if not relieved, determined to deliver her. I called for Dr. White who was spending the night in the house, and while he administered a small amount of chloroform I introduced my hand through the placenta, seized the feet and delivered a living, healthy child. I was not over five minutes making the turning and delivery, and not over four ounces of blood were lost by the patient. The tampon controlled the hemorrhage perfectly during the labor, as not over half an ounce of blood was on it when removed. After delivery the foot of the bed was raised and every effort made to revive the patient, but unsuccessfully, for she died two hours after delivery. We were at a loss to account for her death as but a very small amount of blood was lost. The next morning, however, the mystery was solved. The nurse on going to the water closet found it half full of clotted blood, the blood being 10 or 12 inches in depth. The patient, before I was sent for, went to the water closet, and while there, supposing that she was passing urine, passed this large amount of blood. This explained her weak and faint condition during labor, and her death afterwards. Dr. R—— thinks that there is often more blood lost in these cases in this manner than is supposed. There was only a little oozing after delivery which was controlled by ice in the vagina.

Dr. WYCKOFF related a case to which he was called and delivered a five month child, one week ago last Friday. The patient has had two miscarriages before. Several times during this pregnancy she had hemorrhage, at first a week apart, then more frequently, and lately she had bled a little every day. After four hours of pains she miscarried. The placenta was not entirely over the os, but at one side, and presented a remarkable appearance. A portion, one inch in breadth and six inches in length was softened and putrid. This condition of the placenta explained the frequent attacks of hemorrhage during pregnancy. She has been very sick since delivery, having had metritis, peritonitis, &c., but is recovering.

Dr. HAUENSTEIN stated that he had attended two cases of placenta prævia since his last report of his cases. The first one was attended by a midwife for four days before he saw her and had lost a large amount of blood. She was very feeble, uterus considerably dilated; on examination found the os covered by the placenta. The head had not engaged so I detached the placenta, ruptured the membrane and delivered with the forceps. The placenta in this case was not centrally implanted over the os, but more to one side. The second case was complete placenta prævia. I turned and delivered. There is great danger in these cases from post-partum hemorrhage, owing to the lower part of the uterus not contracting sufficiently to close the mouths of the uterine blood vessels.

Dr. MINER had seen, since the interesting report of so many cases by Dr. Hauenstein, one case of *placenta prœvia* in connection with Dr. Mixer. It was a rare, and to him up to that time, an unheard of placenta; the entire womb was covered by placenta, the os the same as elsewhere. There was hardly to be found any membranous portion, a small place at the superior part was thin and seemed like the usual membrane. Over the entire surface it was placental and evidently attached throughout to the walls of the uterus. The case resulted favorably to both mother and child. Child was turned and delivered after waiting for the os uteri to become fully dilated. Dr. Miner also spoke of a case of midwifery he had recently attended where labor was natural and at full period. The mother was remarkably well for the first three weeks when she commenced to complain of pain in back and lower bowels. Thinking she had been too resolute and up too much, he advised horizontal position and rest. Next morning there was hemorrhage with expulsion of larges masses of coagulated blood. He regarded this as an unusual occurrance at that length of time after delivery.

Dr. GAY remarked that the case of Dr. Rochester was interesting and the treatment proper. He, however, thinks that turning by introducing the hand into the uterus through the placenta, is not the best method of procedure in the majority of cases of placenta prævia. He believes that the method advocated by Barnes of London, when the head presents is best; the placenta should be detached, the membranes ruptured and forceps applied, and the delivery at once effected. This at once stops all hemorrhage. There is most danger of post partum hemorrhage in these cases. Was called a few years since to attend a case of placenta prævia with Dr. Storck; delivered by turning, but the patient afterwards died of peritonitis. Thinks that when a patient has lost a large quantity of blood before being seen, that the bleeding should be controlled and time given for the patient to rally before attempting delivery.

Dr. CRONYN thinks that there is no doubt that death in the majority of these cases results from hemorrhage. He would tampon and apply a bandage over the fundus of the uterus and thereby have complete control of any hemorrhage, and when the os was sufficiently dilated or dilatable would introduce the hand through the placenta and deliver by turning. This method has the sanction of Simpson. Barnes' method cannot always be employed. It is easy to turn when the waters are unruptured. Delivering with forceps as Dr. Gay recommends will answer when the head presents, but in these cases would use tampon and bandage to control hemorrhage. Every case in fact has its appropriate treatment. When one side of placenta has been detached would give quinine with ergot. Has lately been using quinine instead of ergot and has been pleased with the result.

Scarlatina, Typhoid fever and derangements of the bowels, were reported as prevailing.

Adjourned.

W. C. PHELPS, *Secretary.*

